# Assessment of Vitamin D Levels in Patients Presenting With Chronic Low Back Pain at a Tertiary Care Hospital

**DOI:** 10.7759/cureus.11867

**Published:** 2020-12-03

**Authors:** Mukesh Kumar, Masroor Ahmed, Ghulam Hussain, Muhammad Bux, Naveed Ahmed, Sunil Kumar

**Affiliations:** 1 Orthopedic Surgery, Begum Haji Yousuf (BHY) Jamiyiat Hospital Karachi, Karachi, PAK; 2 Orthopedic Surgery, Shaheed Mohtarma Benazir Bhutto Medical College, Karachi, PAK; 3 Orthopedic Surgery, Sheikh Zayed Taluka Headquarter Hospital, Karachi, PAK; 4 Orthopedic Surgery, Khairpur Medical College, Sindh, PAK; 5 Trauma and Orthopedic Surgery, Dow University of Health Sciences, Karachi, PAK

**Keywords:** vitamin d, chronic low back pain (clbp), vas for pain

## Abstract

Objective

To evaluate the association of chronic low back pain with levels of vitamin D in the affected population.

Methodology

This observational study was carried out from August 2016 to August 2019 at Khairpur Medical College and Shaheed Mohatarma Benazir Bhutto Medical College, Karachi, Pakistan. Patients aged 18 years and above suffering from chronic low back pain with pain persisting for more than 12 weeks were the study participants after written consent and prior approval from the ethical review committee was obtained for conducting the study. Data was recorded on predesigned performa and analyzed on SPSS Version 20 (IBM Corp.).

Results

There were 1,152 cases with chronic lower back pain, of whom 632 (54.9%) were females and 520 (45.1%) were males. The mean age of the patients was 41.76 ± 11.18 years. The mean visual analog scale (VAS) level was 5.36 ± 1.65; 707 cases (61.4%) had moderate pain according to VAS, 292 (25.3%) had severe pain, and 153 (13.3%) had mild pain. Concerning vitamin D levels, the mean levels were 22.74 ± 13.80, with 599 (52%) of the patients having deficient levels of vitamin D, 347 (30.1%) having insufficient levels, and only 204 (17.7%) of the cases having normal vitamin D levels.

Conclusions

Lower back pain is one of the common presenting problems in orthopedic clinics. We found no relationship between chronic lower back pain and vitamin D levels in our study.

## Introduction

Deficiency of vitamin D is commonly present around the globe, regardless of developed or developing regions, especially in South East Asia [1], and is considered a global epidemic [2]. Vitamin D deficiency has been present in men and women throughout the world's different communities over a wide range (20-100%) [3]. Most people do not have any of the symptoms, and presentation depends on the grading and duration of vitamin D deficiency [4]. Low back pain is the most common presenting musculoskeletal problems, leading to absence from the duties, increased disability, and socioeconomic cost [5]. Low back pain is labeled as chronic when it lasts for a minimum of 12 weeks [6]. The definitive cause of chronic low back pain is poorly known [7]. Correlation of deficient vitamin D levels with chronic low back pain has been studied, but definitive evidence is still lacking [8]. Endocrine society graded the levels of vitamin D levels into different categories: 20 ng/mL levels or less imply a deficient state, levels from 21 to 29 ng/mL are labeled as insufficient, and levels more than 30 ng/mL are labeled as normal [3]. These levels apply to both children and adults, and levels in the range of 40-60 ng/mL are considered optimum and a maximum of 100 ng/mL is known to be safe levels of vitamin D [9].

This study's main aim was to assess the association of vitamin D levels with chronic lower back pain in patients having pain persisting for more than 12 weeks.

## Materials and methods

This observational study was conducted between August 2016 to August 2019 at Khairpur Medical College and Shaheed Mohatarma Benazir Bhutto Medical College, Karachi, Pakistan. Non-probability consecutive sampling technique was used for the sampling. Patients aged 18 years and above suffering from chronic low back pain persisting for more than 12 weeks who presented for the management of chronic low back pain at our outpatient department were included in the study. Informed written consent was obtained from the participants, and prior approval from the Ethical Review Committee for conducting the study was taken.

Vitamin D levels were measured from the venous blood of the patient. The association of chronic lower back pain with levels of serum vitamin D was evaluated. Data were recorded on a predesigned performa and were entered into IBM SPSS Version 20 (IBM Corp., Armonk, NY, USA) for analysis. Mean and frequencies were recorded for the age, gender, marital status, duration of symptoms, visual analog scale (VAS) for pain, and levels of vitamin D. Stratification of data was performed for gender and vitamin D levels. Pearson's correlation was used to assess the correlation of vitamin D levels with chronic low back pain.

## Results

Descriptive statistics are given in Table 1.

**Table 1:** Descriptive statistics

N = 1,152	Mean ± SD/Frequency
Age	41.76 ± 11.18
Gender	
Male	520 (45.1%)
Female	632 (54.9%)
Marital status	
Unmarried	203 (17.6%)
Married	818 (71.0%)
Divorced	131 (11.4%)
Duration of symptoms (weeks)	22.23 ± 5.88
BMI group	26.01 ± 4.35
Normal (1.8-24.9)	495 (43.0%)
Over-weight (25-29.9)	431 (37.4%)
Obese (>30)	226 (19.6%)
VAS	5.36 ± 1.65
Vitamin D levels	22.74 ± 13.80

**Figure 1:**
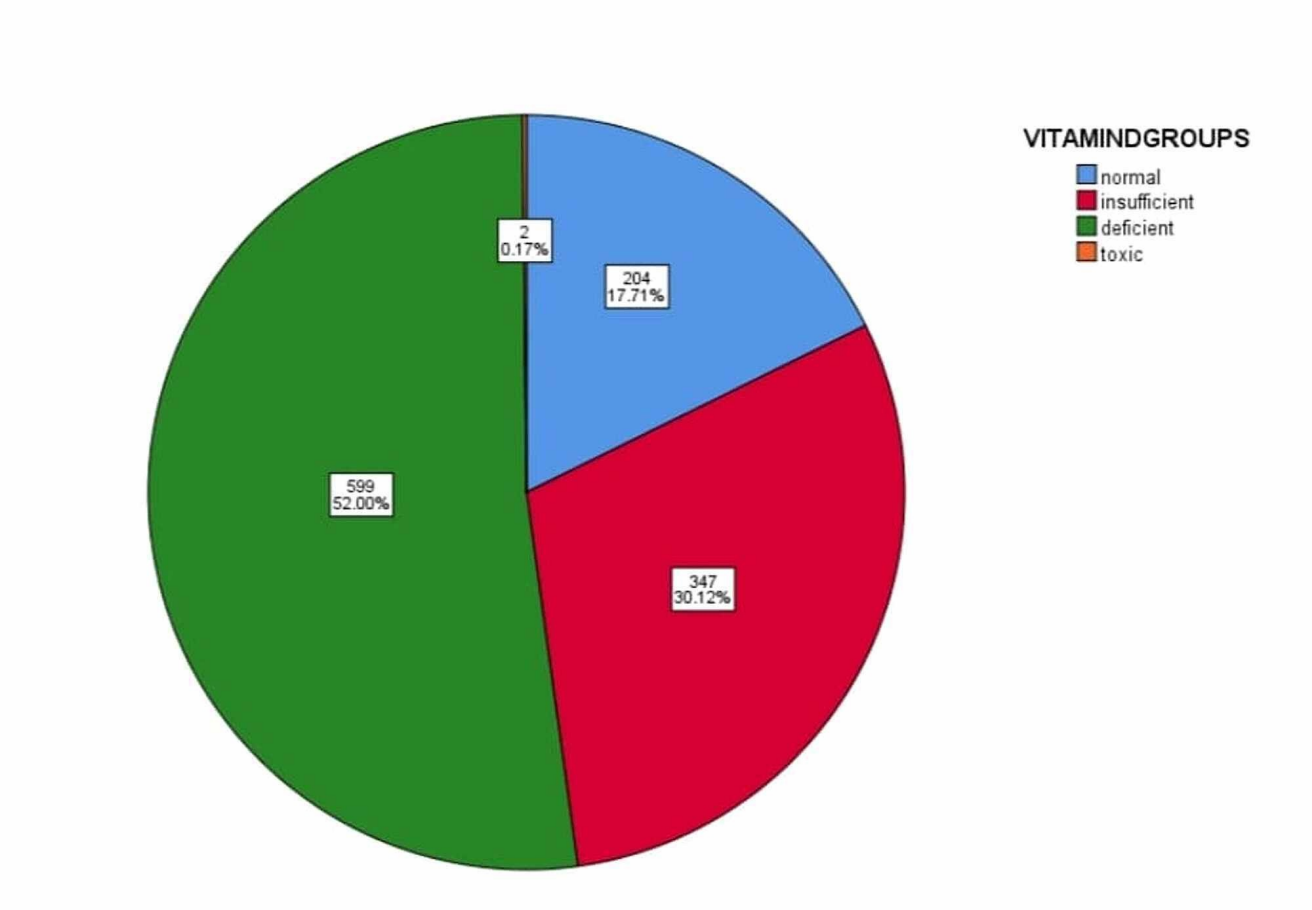
Stratification of data for grading of vitamin D levels.

A total of 1,152 cases presenting with lower back pain were included in the study. The mean age was calculated as 41.76 ± 11.18 years, with a minimum of 18 years and a maximum of 65 years. Concerning gender, 632 were females (54.9%) and 520 (45.1%) were males. Concerning marital status, 818 (71%) of the cases were married, 203 (17.6%) were unmarried, and 131 (11.4%) were divorced. The mean duration of symptoms was 22.23 ± 5.88 weeks. In stratification concerning BMI, the mean levels were 26.01 ± 4.35; 495 (43%) cases were in the normal range, 431(37.4%) were over-weight, and 226(19.6%) were obese. The mean level of VAS for pain was 5.36 ± 1.65, with 707 cases (61.4%) in the moderate pain category according to VAS for pain, 292 (25.3%) in the severe pain category, and 153 (13.3%) in the mild category of pain. In stratification of data concerning vitamin D levels, the mean levels were 22.74 ± 13.80 ng/mL. Out of 1,152 patients, 599 (52%) patients had deficient vitamin D levels and 347 (30.1%) had insufficient levels. Normal levels were observed in 204 (17.7%); two patients had toxic levels of vitamin D, i.e., above 100 ng/mL, as shown in Figure 1.

In stratification of vitamin D levels concerning gender, out of the 520 male patients, 275 cases had deficient levels. Insufficient and normal levels were seen in 159 and 86 cases, respectively. Out of 632 females, normal, insufficient, and deficient levels were observed in 118, 188, and 324 females, respectively, as shown in Table 2.

**Table 2:** Stratification of data for gender and vitamin D levels

		Vitamin D group				Total
		Normal	Insufficient	Deficient	Toxic	
Gender	Male	86	159	275	0	520
	Female	118	188	324	2	632
Total		204	347	599	2	1,152

According to VAS for pain and the stratification of VAS concerning gender, out of 520 cases, 133 had severe pain, 311 had a moderate intensity, and 76 had mild intensity pain. Out of 632 females, severe pain was observed in 159 cases, whereas moderate and mild pain, according to VAS, was observed in 396 and 77 cases, respectively. Pearson's correlation was used to examine the correlation between chronic low back pain measured by VAS for pain and vitamin D levels. We found no correlation between low back pain and vitamin D levels (r = 0.013; p = 0.652) in patients presenting to an orthopedic clinic for the evaluation of low back pain.

## Discussion

Vitamin D is one of the fat solvent hormones, the most critical work of which is the hemostasis of calcium [10]. Vitamin D deficiency presents in different ways, ranging from nonspecific musculoskeletal pain to definitive clinical presentation of osteomalacia such as pain, tenderness, weakness of muscles, and even difficulty in walking [11,12]. The decreased vitamin D levels influence chronic low back pain in our body [12]; decreased levels may also lead to increased pain sensitivity and diminished neurological and muscular functional activity [13]. Deficient levels of vitamin D raise the chances of inflammatory activity at the vertebral endplates, causing the diminished pain threshold and thus resulting in generalized pain in the muscle and bone leading to weakness [14]. Few studies assessed the association between vitamin D and intensity of pain, and some pointed out a strong correlation between both the variables [15].

In contrast, others, such as Johansen et al. [16] and Ghai et al. [17], showed no definitive association between vitamin D levels and pain intensity. Another study by Hicks et al. showed a correlation between low vitamin D levels and backache in the female population [18]. Our study tried to determine the relationship between chronic low back pain and vitamin D level, and we found no correlation between vitamin D and low back pain. In our study, about 82.11% of the cases had abnormal (insufficient and deficient) vitamin D levels, and only 17.71 had normal levels. In a study by Gokcek and Kaydu, 85.7% of the cases had abnormal levels of vitamin D, and normal levels were observed in 14.3% [19]. In a similar study by Karahan et al., 87.5% of the total cases had abnormal vitamin D levels, and 12.4% had a normal range of vitamin D [20]. The results of the two aforementioned studies were almost comparable with our study in terms of the abnormal levels.

## Conclusions

There was no correlation between chronic low back pain and levels of vitamin D in our study. Still, despite that, around 81.01 % had abnormal levels of vitamin D. Based on this finding, it can be stated that all the patients presenting with chronic low back pain need evaluation of vitamin D, as it may be the potential cause of pain in patients.
